# The search for instantaneous vection: An oscillating visual prime reduces vection onset latency

**DOI:** 10.1371/journal.pone.0195886

**Published:** 2018-05-23

**Authors:** Stephen Palmisano, Bernhard E. Riecke

**Affiliations:** 1 School of Psychology, University of Wollongong, Wollongong, New South Wales, Australia; 2 School of Interactive Arts and Technology, Simon Fraser University, Vancouver, British Columbia, Canada; University of Muenster, GERMANY

## Abstract

Typically it takes up to 10 seconds or more to induce a visual illusion of self-motion (“vection”). However, for this vection to be most useful in virtual reality and vehicle simulation, it needs to be induced quickly, if not immediately. This study examined whether vection onset latency could be reduced towards zero using visual display manipulations alone. In the main experiments, visual self-motion simulations were presented to observers via either a large external display or a head-mounted display (HMD). Priming observers with visually simulated viewpoint oscillation for just ten seconds before the main self-motion display was found to markedly reduce vection onset latencies (and also increase ratings of vection strength) in both experiments. As in earlier studies, incorporating this simulated viewpoint oscillation into the self-motion displays themselves was also found to improve vection. Average onset latencies were reduced from 8-9s in the no oscillating control condition to as little as 4.6 s (for external displays) or 1.7 s (for HMDs) in the combined oscillation condition (when both the visual prime and the main self-motion display were oscillating). As these display manipulations did not appear to increase the likelihood or severity of motion sickness in the current study, they could possibly be used to enhance computer generated simulation experiences and training in the future, at no additional cost.

## Introduction

Our movements through the world are registered by vision, audition, the vestibular system of the inner ear, the somatosensory system of cutaneous receptors, and the proprioceptive system of muscle and joint receptors [1,2]. While the stimulation of any of these senses can generate a perception of self-motion, visual motion stimulation appears to play a particularly important role (e.g. [3], see [4] for a recent review). Indeed it has long been known that compelling illusions of self-motion can be induced in physically stationary observers by visual stimulation alone [5–6]. These visual illusions of self-motion have been traditionally termed ‘vection’ (although see [7] for the history of this term and other possible self-motion related uses of ‘vection’). While this vection has been extensively studied in the laboratory (see [8,9] for reviews), it can also occur in everyday life. For example, vection is often experienced when one is sitting on a stationary train and then observes another train on an adjacent track departing the station [10,11].

Vection is now also an increasingly common experience during first person virtual reality (VR) and vehicle simulation [12–15]. In fact, the experience appears to be difficult to avoid in modern head-mounted display (HMD) based gaming (since these devices now cover fields of view which are greater than 60 degrees in visual angle and vection is known to increase with the area of the retinal motion stimulation [8–9]). It has been argued that vection during VR increases the likelihood of visually induced motion sickness (VIMS) (e.g. [16]). However, the empirical support for such a relationship between vection and VIMS is mixed (see [17] for a recent review). By contrast, evidence is now beginning to emerge that vection could provide functional benefits during computer-generated self-motion simulation. Vection has been reported to increase the realism and believability of these simulations [18]. In addition, research suggests that vection can also increase presence, improve spatial updating, reduce disorientation and promote greater user/gamer involvement [9,18–25].

For vection to be most useful/beneficial in VR and vehicle simulation it should be induced quickly, if not immediately [26–27]—since delays between the start of display motion and the visually induced experience of self-motion will alter the way in which users act in, and interact with, their virtual environment. Unfortunately, when an external optic flow display is presented to a stationary observer there is often a delay of up to 10 s or more before vection is first reported [28,29]. This vection onset latency can however be considerably longer (e.g. [30]) and appears to depend on a variety of factors (including the vection inducing potential of the display, as well as the characteristics of the observer) [31–33]. Similar delays in vection induction are also common when this visual motion stimulation is presented via HMDs [34,35]. In both cases, the observer will typically first perceive object motion, then combined object-and-self-motion, and finally (assuming favourable conditions) exclusive self-motion. The initial vection onset latency is generally thought to reflect the time required to resolve sensory conflicts generated by the observer’s visual and non-visual inputs [36]. When a person is stationary during seated VR gaming and fixed-base vehicle simulation, the experience of their visually simulated self-motion will not be supported by their available non-visual information. According to sensory conflict theories, the onset of vection will be delayed in these situations because the expected vestibular stimulation is absent (i.e., there is no confirmation from the inner ear that the observer has indeed accelerated up from being stationary to the visually simulated speed of self-motion [36]). However, an alternative explanation for this empirically observed vection onset latency might be that it represents the time taken to suppress the default visual processing responsible for object motion, prior to the induction of vection [37,38].

While immediate (or nearly immediate) vection would appear to be the ideal for first person VR and vehicle simulation applications, it has proved difficult to substantially reduce vection onset latencies in stationary observers [7,9,39]. Consistent with the sensory conflict accounts described above, evidence suggests that vection onset latency can be reduced by physically moving the observer’s body to corroborate his/her available visual motion information. Research has shown that vection onset latency can be: (1) significantly reduced by brief physical observer motions in the direction expected for the visual simulation [31,40–44]; and (2) delayed by providing conflicting visual and vestibular stimulation [45–47]. In a similar fashion, galvanic vestibular stimulation can also be used to enhance vection if it is reasonably compatible with the available visual motion stimulation. For example, applying galvanic vestibular stimulation during visual motion has been found to reduce vection onset latencies and increase vection strength [27,48]. Applying bone conducted vibration at the mastoid processes (known to affect the vestibular system) also appears to have a similar effect on vection onset latency. For example, this type of vibration was found to reduce the delay in vection onset latency by 2 s in a recent study by Weech and Troje [27]. These authors proposed that this vibration reduces vection onset latency because it triggers a sensory readjustment (i.e., vestibular down-weighting) to both reduce visual-vestibular conflict and favour visual self-motion perception.

As noted above, physical observer motions are often not possible in VR or during vehicle simulation. While galvanic and other types of vestibular stimulation have the potential to reduce vection onset latencies in future applications [48–50], at the moment they are rather coarse, invasive and uncomfortable (as discussed by [51]). Visual display manipulation may therefore present a more practical and affordable solution to the problem of reducing vection onset latencies in the near future. For example, adding stereoscopic information to self-motion displays has been shown to reduce vection onset latencies (compared to non-stereoscopic and monocularly viewed display conditions—see [52–55]). Research has also shown that vection onset latencies can be significantly reduced by adding simulated viewpoint oscillation to visual self-motion displays [4,56–67]. The result of adding this simulated viewpoint oscillation to vection-inducing displays is similar to the visual effects of bob and sway head movements seen during real-world walking [4] (most past studies on vection have focussed on simulating bob and sway head-motions, only a handful have examined viewpoint jitter/oscillation along all three axes [58,66]).

In the present study we will examine whether presenting an oscillating visual priming stimulus just before a forward visual self-motion display can further reduce, or even better remove, the onset latency for vection in depth (compared to control conditions, where observers instead view a static visual prime before each self-motion display). The oscillating prime stimulus will only simulate sideways horizontal and vertical viewpoint oscillation relative to the 3D virtual environment, and thus will always be perpendicular to the subsequently simulated forward self-motion. The priming stimulus will be followed by the presentation of either a smooth or an oscillating pattern of radially expanding optic flow. Both of these vection displays will simulate the same amount of forwards self-motion in depth. However, the oscillating radial flow will also simulate horizontal and vertical viewpoint oscillation as well (the oscillating component of this optic flow pattern will be identical to that provided in the initial priming display). As noted above, despite the assumed increase in sensory conflict generated by adding simulated viewpoint oscillation, oscillating patterns of radial flow actually induce superior experiences of vection in depth than smooth patterns of radial flow (as indicated by stronger vection ratings, earlier vection onsets and longer cumulative durations of vection—see [4] for a review of this literature). In this study we will examine whether providing an oscillating visual prime directly beforehand will: (1) further reduce the vection onset latencies for oscillating patterns of radial flow; and (2) also reduce the vection onset latencies for smooth patterns of radial flow (compared to when these two types of radial flow were preceded by a static visual prime).

### Why might an oscillating prime reduce vection latency?

There are several reasons why presenting an oscillating visual prime before radially expanding patterns of optic flow might reduce vection onset latency.

#### Direct vection induction by prime

The first possibility is that the oscillating prime itself might induce (horizontal and vertical) oscillatory vection prior to any exposure to the radially expanding flow (and therefore the induction of vection in depth). Support for this hypothesis is provided by earlier research which examined the simulated viewpoint jitter advantage for vection (first reported by Palmisano, Gillam & Blackburn [68]). Simulated viewpoint jitter is quite similar in both its appearance and effects on vection to simulated viewpoint oscillation, with the main difference being that it simulates random (as opposed to periodic) observer head motions [4]. Previously Palmisano, Burke and Allison [69] compared the vection induced by presenting horizontal and vertical simulated viewpoint jitter alone to the vection induced by smooth and jittering patterns of radial flow. While the jitter-only condition was found to induce some vection on its own, it was far from optimal—starting much later and lasting for a shorter time than the vection induced by the jittering and non-jittering radial flow. Based on these findings, it is therefore possible that the oscillating prime stimulus might also induce weak horizontal and vertical vection in the current study (even though there is new evidence which suggests that these simulated viewpoint jitter and viewpoint oscillation effects might have different origins—see studies [57,67]).

#### Sensory readjustment

A second possibility is that prior exposure to simulated viewpoint oscillation might trigger a sensory readjustment so as to favour vection induction. According to sensory conflict theory, simulated viewpoint oscillation should produce significant and sustained visual-vestibular conflict in stationary observers (see [4] and [36]). However, it is likely that conflicting vestibular inputs (indicating stationarity) might be down-weighted during prolonged exposure to this visual oscillation (so as to reduce the level of visual-vestibular conflict over time). This should prime observers towards visual motion and increase the vection inducing potential of any subsequently presented optic flow. A recent study by Seno and colleagues [70] provides indirect support for this notion. They found that prior walking without optic flow transiently delayed the subsequent induction of vection from optic flow (compared to the vection induced after an equivalent period of walking with normal vision). It was concluded that walking without optic flow triggered a down-weighting of visual (relative to non-visual) self-motion information, which reduced the ability of optic flow to induce self-motion perception in physically stationary observers. Another study by Weech and Troje [27] concluded that vection onset latencies were reduced via a different type sensory readjustment (in their study adding vestibular noise was argued to favour visual vection induction by triggering vestibular down-weighting). Taken together these studies suggest that it might be possible for a purely visual stimulus to alter the experience of subsequently induced vection (even if the oscillating prime itself could not induce vection on its own).

#### Increased sensitivity to global optical flow

A third, but related, possibility is that the oscillating prime might reduce vection onset latency by sensitizing observers to global patterns of optic flow. The findings of Ito [71] provide support for this hypothesis. His study found that changing the direction of the simulated self-motion during a trial caused a dropout in the vection induced by the original optic flow pattern. However, while there was still a delay in inducing the new/second simulated direction of self-motion, it was 15% less than the vection onset latency for the original direction of self-motion. This finding suggests that prior exposure to global motion can sensitize observers to self-motion compatible patterns of optic flow and thus prime them to experience vection more quickly in the future. This finding also suggests that even if participants in our study sometimes experience weak vection during the oscillating prime, it should not transfer directly to any subsequently presented radial flow (which would simulate an orthogonal direction of self-motion compared to the priming stimulus). In fact, Ito [71] state that “vection was lost when the direction of the flow changed, and that vection in a new direction requires a new latency period” (p. 35).

The primary purpose of this study was to investigate whether visual priming with simulated viewpoint oscillation can reduce or even remove vection onset latencies for subsequently presented displays which simulate self-motion in an orthogonal direction (and perhaps also increase the strength of the vection experience as well). The three perceptual hypotheses outlined above build the case for conducting such an investigation. If, as hypothesised, visual priming is found to benefit the vection induced by large external displays in Experiment 1, then Experiment 2 will test whether these effects also generalise to a different (HMD based) display system, and Experiment 3 will examine the possible mechanism/s responsible such effects. The hypothesized priming effects could have practical implications for the future of VR, tele-presence/tele-operation, and vehicle simulation by providing more compelling and affordable self-motion simulations. However, this would only be the case if the display manipulations used did not increase the likelihood of experiencing motion sickness. Accordingly, this study also examined the effects of the visual oscillation on the likelihood and severity of participants experiencing VIMS.

## Experiment 1: Vection induced by a large, distant external display

Experiment 1 examined the effects of prior presentation of an oscillating (versus a static) visual prime on the vection induced by smooth and oscillating patterns of radial flow. In this experiment, both the priming and the main self-motion displays were projected onto a large distant external screen. This experimental setup is typical of many laboratory-based experiments on vection (e.g. [62–67]) and similar to the stimulation provided by certain types of fixed-based vehicle simulation.

### Methods

#### Participants

Sixteen female and seven male psychology students (mean age = 21.8 years, *SD* = 3.4 years) from the University of Wollongong participated in this experiment. They all had normal or corrected-to-normal vision and were naïve to the experimental hypotheses. They had not experienced vection in the laboratory prior to this experiment and all reported feeling well at the start of the experiment. When questioned they reported having no existing vestibular or neurological impairments. The University of Wollongong ethics committee approved the study in advance (HE10/120) and each participant provided written informed consent before participating in the study.

#### Apparatus

Self-motion displays were generated by a DELL OPTIPLEX 9020 personal computer with an AMD Radeon HD 8570 video card. A NEC (Model NP—P401WG) LCD data projector (1280 x 1024 pixel resolution with a refresh rate of 60 Hz) was used to front project these displays onto a large 5.0 m wide by 2.6 m high screen. Participants were seated 3.5 m in front of the screen and viewed these displays through a viewing booth that blocked all stationary room features from view. As a result, each display subtended a 71 degree wide by 41 degree high visual area at the observer’s eye. When viewing these self-motion displays vection onset latency and duration responses were recorded using button presses on a Dell M0C5U0 USB Scroll 3 Button Optical Mouse. This mouse rested on a table in front of the seated participant (who placed one hand on the mouse and rested the other hand on the table). Verbal vection strength ratings were manually entered on the computer’s keyboard by the experimenter after each display.

#### Design

Three independent variables were manipulated in this experiment: (1) Prime Type. Displays initially simulated that the observer was either stationary (static prime) or oscillating up-and-down as well as from left-to-right (oscillating prime) relative to the 3-D virtual environment. (2) Radial Flow Type. After 10 s exposure to the priming stimulus, these displays then began to simulate self-motion in depth. They either simulated smooth forwards self-motion (smooth radial flow) or forwards self-motion combined with simulated horizontal and vertical viewpoint oscillation (oscillating radial flow). (3) Block. There were 4 blocks of experimental trials. Each block presented the four different Prime Type by Radial Flow Type conditions in a different random order. Five different dependent variables were obtained for each experimental trial: (1) the time from the start of the display motion until vection was first experienced (i.e., the vection onset latency in seconds); (2) the total duration of their experience of vection (in seconds); (3) a verbal rating of the overall vection strength experienced during these displays (from 0–10); (4) a yes/no response to the question ‘do you feel sick?’; and (5) a verbal rating of the severity of the sickness experienced at the end of the trial (from 0–20; using the Fast Motion Sickness (FMS) scale by Keshavarz & Hecht [72]).

#### Visual motion stimuli

The standard stimulus for this experiment was a 30-second radial flow display which simulated constant velocity forwards self-motion at 1.1 m/s. Display durations were 10-seconds longer in the practice and experimental trials which followed. Each of these trials initially presented a 10-second priming display, followed by a 30-second forward self-motion display. An auditory countdown was played during the priming phase (from 10 down to 1) to warn participants about the upcoming transition to the radial flow phase. Both the prime and flow displays simulated a 3-D cloud environment consisting of 3000 blue circular objects on a black background (cloud dimensions were 12.3 m wide × 5.8 m high and 13.1 m deep; objects were simulated to be 3.5 cm in diameter). During the 10-second priming phase of the trial, displays suggested that the participants were either stationary or moving (up-and-down as well as from left-to-right) relative to this 3D cloud. Then during the subsequent 30-second radial flow phase of the trial, these displays began to simulate forwards self-motion in depth (via global motion perspective cues and local changes in object image sizes; no stereoscopic cues to motion in depth were provided). During this radial flow phase, there was initially a 1-second period of acceleration up from stationary to the constant simulated forward speed of 1.1 m/s. The simulated forward self-motion remained at 1.1 m/s for the next 29-seconds, after which all display motion ceased and the objects disappeared. In half of the experimental trials, displays simulated horizontal and vertical viewpoint oscillation as well as this self-motion in depth. When it was present in either the priming or radial flow display phases, this simulated viewpoint oscillation had amplitudes and frequencies of 4.4 cm and 1 Hz along the observer’s horizontal axis, and 2.2 cm and 2 Hz along his/her vertical axis. The motion of the simulated viewpoint due to this oscillation traced out a path in the frontal plane similar to a figure-eight pattern lying on its side. These oscillation frequencies and amplitudes were selected based on iterative testing (and formative evaluations) in pilot studies. They were also chosen to be compatible with VR/gaming applications. Our aims were to avoid sharp accelerations (that might be too nauseogenic) and to mimic subtle head-oscillations (similar to head-bobbing when listening to music). The luminance of the objects in these displays ranged from 0.2 (min) to 1.5 cd/m^2^ (max) and they were presented on a 0.15 cd/m^2^ black background.

#### Procedure

Participants were told that in the main experiment each trial would consist of a 10-second priming display (which would either be static or oscillating), followed by a 30 s display simulating forwards self-motion in depth. They were also told that: (1) sometimes the objects might appear to be moving and at other times they might feel as if they were moving themselves (relative to the objects); and (2) we were interested in when they actually felt that they were moving. Participants were told that they could press a button at any time during each 40s trial to indicate perceived self-movement. At the beginning of the experiment, participants were first shown the standard stimulus display (a radially expanding pattern of optic flow) which was used to set the modulus for their subsequent vection strength ratings (as per the method of magnitude estimation—Stevens [73]). After viewing this display, they were asked whether they had felt that they were moving or not. If they reported experiencing self-motion they were told that their experience of illusory self-motion should be rated as a ‘5’ (with ‘0’ representing no feeling of self-motion). If a participant did not experience vection on their first exposure to optic flow, they were re-exposed to the standard stimulus display and also to an oscillating pattern of radial flow. All participants eventually experienced vection (None were excluded from the study). Participants were next presented with four practice trials, where each of the displays had both a 10-second priming phase and a 30-second radial flow phase. During these trials, participants were told that they should press the left mouse button at any time as soon as they felt that they were moving and hold it down as long as their experience of self-motion continued. At the end of each trial, participants verbally rated the overall strength of their vection experience (with ‘0’ indicating no perceived self-motion and ‘10’ indicating very strong perceived self-motion). They were also asked whether they felt sick or well, and then rated their sickness level from 0 to 20 (with 0 indicating “no sickness” and 20 indicating “severe/frank sickness”). There was a 60 s rest period between trials. After completing the practice trials, participants were presented with the four blocks of experimental trials. The 30-second standard stimulus was again presented at the beginning of each block of trials. Then the following four experimental trials were presented in random order: (1) static prime followed by smooth radial flow (S1 Demo Movie); (2) static prime followed by oscillating radial flow (S2 Demo Movie); (3) oscillating prime followed by smooth radial flow (S3 Demo Movie); and (4) oscillating prime followed by oscillating radial flow (S4 Demo Movie).

## Results

Separate 2 (Prime Type: Smooth or Oscillating) x 2 (Radial Flow Type: Smooth or Oscillating) x 4 (Block: 1–4) repeated measures analyses of variance (ANOVAs) were performed on: (1) the vection onset latency data; (2) the vection strength rating data, and (3) the Fast Motion Sickness (FMS) rating data. Greenhouse-Geisser corrections were applied whenever the assumption of sphericity was violated. The data for this experiment are also provided as supplementary materials (see S1 Data for Analysis Experiments 1–3).

### Vection data

Our 23 participants each responded to the 16 experimental trials (four presentations of each the four different display types) as well as the 4 practice trials. Vection was reported on all 460 trials. It should be noted that of these 460 trials, only 11 had negative vection onset latencies (i.e., vection onsets that occurred during the earlier 10 s priming phase). Since vection onset latencies and vection durations were highly correlated, only the vection onset latency and strength data are reported below.

#### Vection onset latency

**Fig 1:**
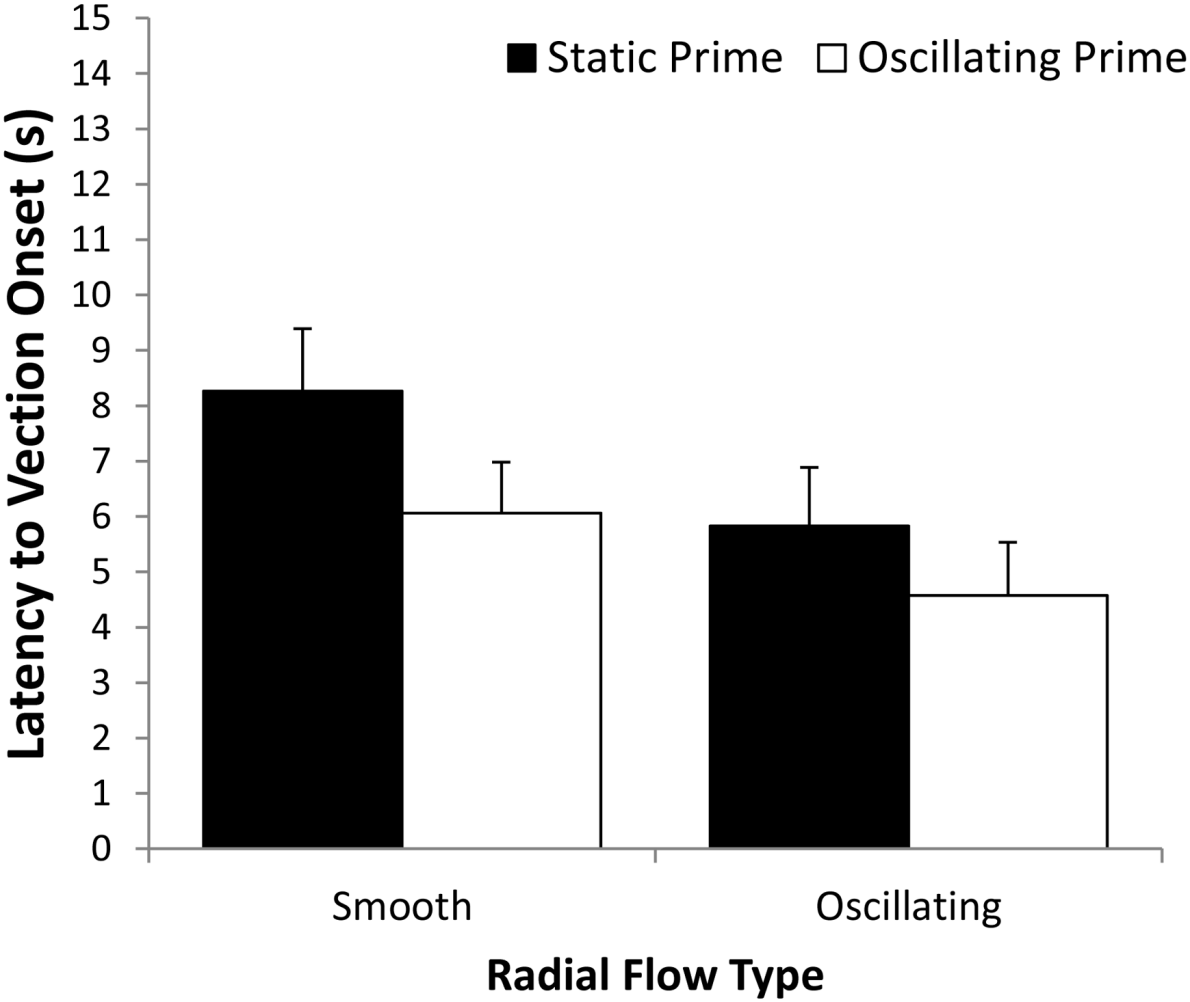
Effects of Prime Type (static or oscillating) on the mean vection onset latencies for smooth and oscillating patterns of radially expanding optic flow. Error bars depict standard errors of the mean (SEMs).

Since it was possible that trials with negative onset latencies represented direct vection induction by the prime itself (rather than a priming effect per se for the subsequent stimulus), all negative onset latencies were replaced with zeroes prior to conducting the repeated measures ANOVA. Eleven trials (2.3% of the total trials) with negative onset latencies were replaced with zeros (2 of these occurred during the practice block). Fig 1 shows the adjusted mean vection onset latencies for each of the Prime Type by Radial Flow Type conditions. We found a significant main effect of Prime Type for vection onset latency, *F*(1,22) = 12.172, *p* = 0.002, partial η^2^ = 0.356. This indicated that oscillating prime conditions (*M* = 5.3 s) produced shorter vection onset latencies than static prime conditions (*M* = 7.1 s). A significant main effect of Radial Flow Type was also found for vection onset latency, *F*(1,22) = 5.797, *p* = 0.025, partial η^2^ = 0.209. This indicated that vection was induced more quickly by oscillating radial flow (*M* = 5.2 s) than by smooth radial flow (*M* = 7.2 s). However, the main effect of Block was not significant for vection onset latency, *F*(1.786,39.291) = 2.936, *p* = 0.07, partial η^2^ = 0.118. Similarly, none of the 2-way or 3-way interactions were found to reach significance. The optimal vection condition (oscillating prime followed by oscillating radial flow) had an average vection onset latency of 4.58 s. A one-way *t*-test on the means of the twenty-two participants in this vection condition indicated that these onset latencies were still significantly greater than zero, *t*_22_ = 4.78, *p* = 0.0001.

We also conducted a more conservative analysis where the negative onset latencies were removed (rather than being replaced by zero as in the above analysis). We calculated the latency data for this more conservative analysis by averaging all of positive latencies across the blocks for each of the conditions. There were still significant main effects of Prime Type {*F*(1,22) = 10.915, *p* = 0.003, partial η^2^ = 0.332} and Radial Flow Type {*F*(1,22) = 5.831, *p* = 0.025, partial η^2^ = 0.210} for vection onset latency. As in the original analysis above, no other main effects or interactions reached significance.

#### Vection strength ratings

**Fig 2:**
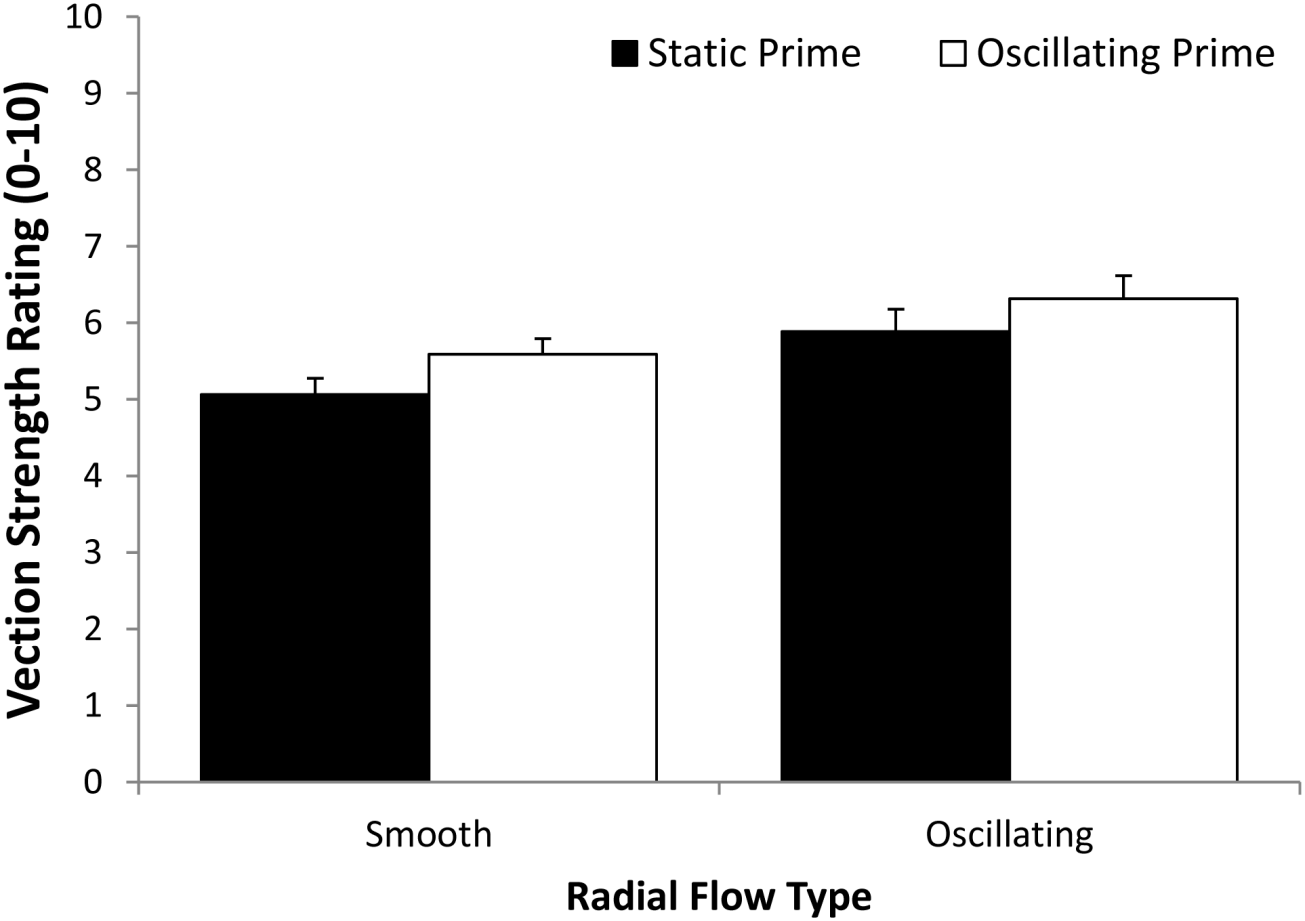
Effects of Prime Type (static or oscillating) on the mean vection strength ratings for smooth and oscillating patterns of radial optic flow. Error bars depict standard errors of the mean (SEMs).

Fig 2 shows the mean vection strength ratings for each of the Prime Type by Radial Flow Type conditions. We also found a significant main effect of Prime Type on vection strength ratings, *F*(1,22) = 24.46, *p* = 0.0001, partial η^2^ = 0.526. This indicated that oscillating prime conditions (*M* = 6.0) generated stronger vection ratings than the static prime conditions (*M* = 5.5). A significant main effect of Radial Flow Type was also found, *F*(1,22) = 12.361, *p* = 0.002, partial η^2^ = 0.360. This indicated that oscillating radial flow (*M* = 6.1) induced stronger vection ratings than the smooth radial flow (*M* = 5.3). The main effect of block was not significant for vection strength ratings, *F*(2.013,44.29) = 1.19, *p* = 0.322, partial η^2^ = 0.051. None of the 2-way or 3-way interactions were found to reach significance.

### Sickness data

#### Sick versus well groupings

**Fig 3:**
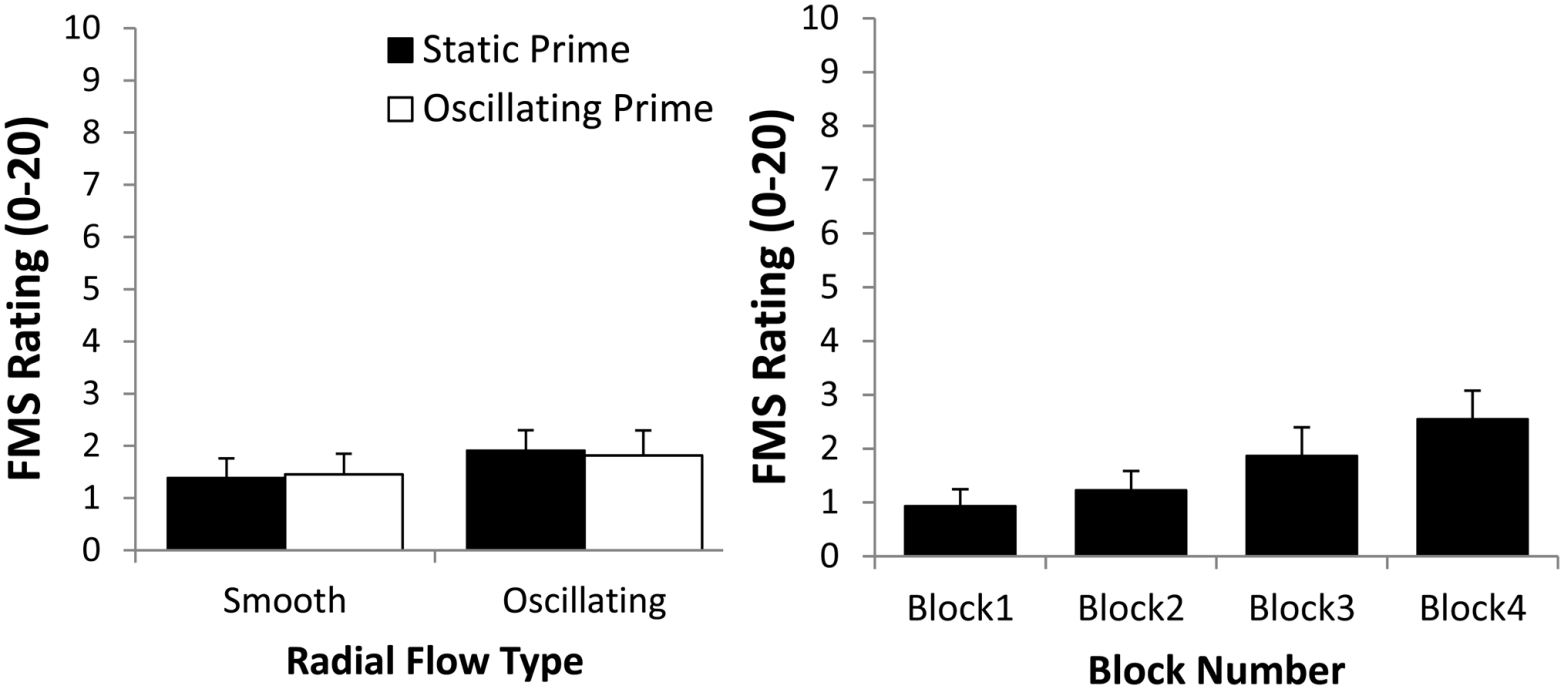
(Left) Mean sickness (FMS) ratings for the eleven ‘sick’ participants for the four different Prime Type by Radial Flow Type conditions (note: the other twelve ‘well’ participants had FMS ratings of zero on all four conditions). (Right) The mean sickness severity ratings for these eleven ‘sick’ participants increased from block 1 to block 4. Error bars depict standard errors of the mean (SEMs).

Participants were classified into ‘well’ or ‘sick’ groups based on their responses to the question ‘do you feel sick?’, which was asked after each trial. The eleven participants who experienced sickness were thus classified as ‘sick’ and the remaining twelve were classified as ‘well’. When sickness was reported, the severity of these symptoms appeared to be quite modest (as can be seen in Fig 3).

#### Sickness severity ratings

Fig 3 shows the mean FMS sickness severity ratings for the eleven participants who were classified as being ‘sick’ (the remaining twelve participants always had FMS ratings of zero). The main effect of Block was found to be significant for FMS ratings, *F*(1.64,36.17) = 5.86, *p* = 0.009, partial η^2^ = 0.210. Overall, FMS ratings increased from a mean of 0.45 for the first block of trials to a mean of 1.22 for the last block of trials (note: the maximum possible sickness rating was 20). However, no other experimental manipulations significantly affected FMS ratings. Neither the main effect for Prime Type (*F*(1,22) = 0.006, *p* = 0.937, partial η^2^ = 0.0001), nor the main effect for Radial Flow Type (*F*(1,22) = 4.170, *p* = 0.053, partial η^2^ = 0.159) were found to be significant (see Fig 3 Right). In addition, none of the 2-way or 3-way interactions reached significance for these FMS ratings.

### Examining the relationship between vection and sickness

#### Sickness and vection strength

**Fig 4:**
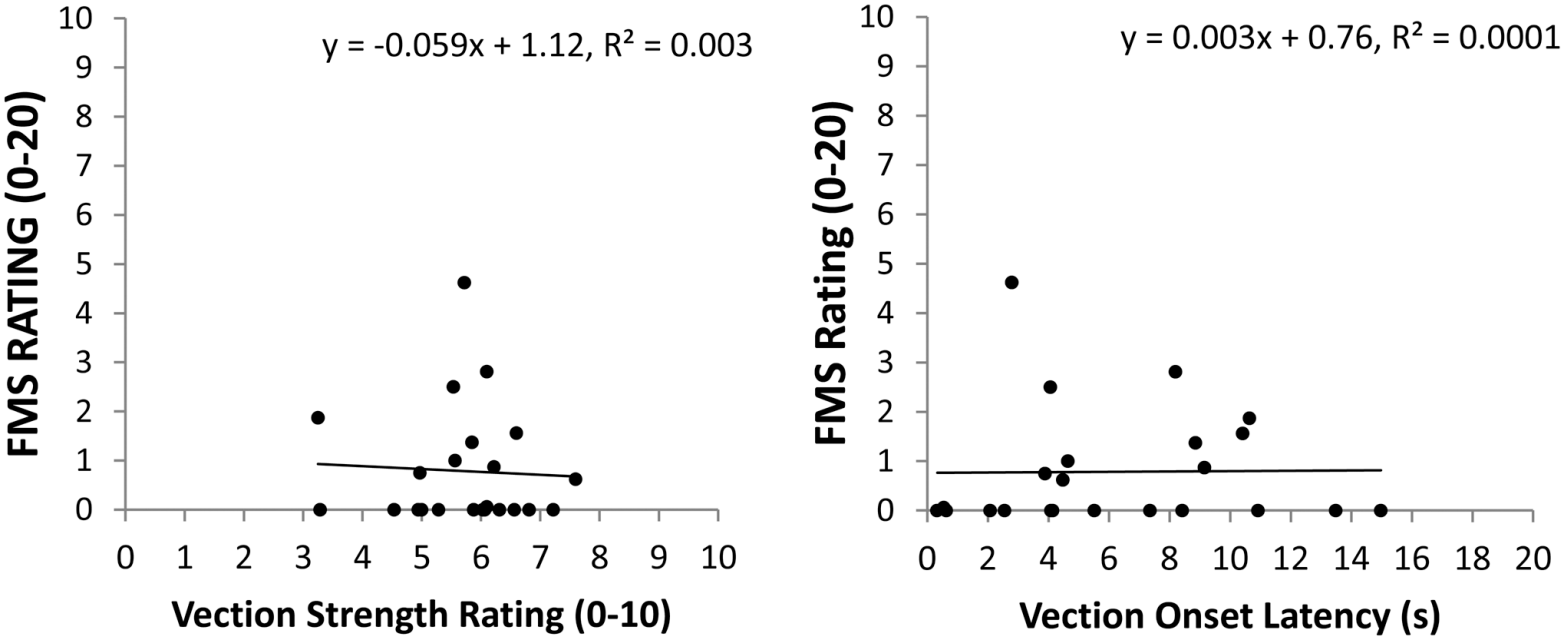
(Left) Plot of mean sickness severity ratings against mean vection strength ratings. (Right) Plot of mean sickness severity ratings against mean vection onset latencies. These plots show the data for both ‘sick’ and ‘well’ participants (the latter had FMS ratings of ‘0’). Relationships between vection and sickness were non-significant and remained so even when only the ‘sick’ participants were included in these analyses.

Vection strength ratings did not predict either the incidence of sickness or the severity of sickness ratings. We first compared the vection strength ratings of participants in the ‘sick’ and ‘well’ groups with an independent samples *t*-test. Participants in the ‘sick’ (*M* = 5.8, *SD* = 1.1) and ‘well’ (*M* = 5.7, *SD* = 1.1) groups did not have significantly different vection strength ratings, *t*_21_ = -0.24, *p* = 0.81. We next performed a linear regression with the participants’ vection strength ratings as the predictors and their FMS ratings as the response variable. However, the relationship between the averaged vection strength ratings and the averaged FMS severity ratings was not found to be significant, *R*^*2*^ = 0.003, *t*_21_ = -0.238, *p* = 0.814 (Fig 4 Left).

#### Sickness and vection onset latencies

Vection onset latencies did not predict either the incidence of sickness or the severity of sickness ratings, mirroring the results for vection strength. We first compared the vection onset latencies of participants in the ‘sick’ and ‘well’ groups with an independent samples *t*-test. Participants in the ‘sick’ (*M* = 6.15 s, *SD* = 3.4 s) and ‘well’ (*M* = 6.2 s, *SD* = 4.9 s) groups did not have significantly different vection onset latencies, *t*_20_ = 0.03, *p* = 0.98. We next performed a linear regression with the participants’ vection onset latencies as the predictors and their FMS ratings as the response variable. However, the relationship between the averaged vection onset latencies and the averaged FMS scores was also not significant, *R*^*2*^ = 0.0001, *t*_21_ = 0.054, *p* = 0.96 (Fig 4 Right). This relationship was found to remain non-significant when only the ‘sick’ participants were included in the analysis, *R*^*2*^ = 0.002, *t*_9_ = 0.12, *p* = 0.904.

## Discussion

This study found that vection onset latencies could be considerably reduced by presenting participants with an oscillating (compared to a static) visual prime lasting only 10s. This oscillating prime also improved vection strength ratings. As in past studies, adding the same oscillation to the subsequently presented radial flow was also found to reduce vection onset latencies and increase vection strength ratings [4]. The vection-enhancing effects of providing oscillation in the two different trial phases appeared to be additive and independent (as indicated by the lack of significant Prime Type by Radial Flow Type interactions). An oscillating prime was still able to improve vection when the subsequently presented radial flow was smooth (as opposed to oscillating). Similarly, even when the priming stimulus was static and the oscillation was only provided during the radial flow, the vection was still superior to that induced by a smooth pattern of radial flow. The oscillating prime followed by the oscillating radial flow condition was found to produce both the fastest vection onsets and the strongest vection ratings of any of the conditions tested. This condition had a vection onset latency (*M* = 4.6 s) which was on average 3.7 s (or 45%) shorter than that for the no oscillation control condition (i.e., the static prime followed by the smooth radial flow).

In this experiment, providing an oscillating (as opposed to static) prime did not significantly increase the occurrence or the severity of reported experiences of motion sickness. While this is promising for the potential future use of such display manipulations in VR and simulation based applications, it is important to note that the durations of visual motion were quite brief (each lasting only 30 to 40 s depending on the particular condition being tested). The sickness severity ratings, while generally modest, were also found to increase significantly over time with repeated exposure to the motion displays. So some caution in interpreting these results is probably warranted.

This experiment found that an oscillating prime and adding oscillation to radial flow could both reduce the onset latencies of the vection induced by a large external display. While vection strength was also increased by these oscillation-based display manipulations, the likelihood of participants experiencing sickness did not appear to be altered. However, we need to determine whether these vection and sickness findings generalize to different display types. HMDs are becoming increasingly affordable and wide-spread. Thus, it would be useful to determine whether oscillating primes and oscillating radial flow can also reduce vection onset latencies with a HMD based display system.

## Experiment 2: Vection induced by a HMD

This experiment investigated whether the vection advantages provided by the oscillating prime in Experiment 1 also generalise to HMD-based self-motion simulations. The HMD we used not only stimulated a significantly larger field of view (100 degrees diagonal) than the external display used previously, but it also allowed us to add consistent stereoscopic cues to both our priming and radial flow displays. Since both an increased field of view and stereoscopic cues have been previously shown to enhance the vection induced by radially expanding optic flow (see [9] for a recent review), it was expected that vection onset latencies would be generally shorter in this follow-up experiment. However, it was also possible that these factors might interact with our oscillation-based display manipulations to increase the likelihood of motion sickness.

### Methods

The stimuli used were similar to those in Experiment 1, apart from simulating a larger field of view (100 degrees diagonal) and also providing consistent stereoscopic cues to self-motion in depth. Participants were told to hold their heads still and look directly forward (to keep the viewing conditions similar to those of Experiment 1). Head tracking was also turned off in this experiment (recent evidence suggests that disabling head tracking alleviates or minimises cybersickness in HMDs [13]). To test for motion sickness in this experiment, we again asked participants whether they felt sick or not at the end of each trial. Unlike Experiment 1 we did not also obtain sickness severity ratings using the FMS scale. However, we still asked participants whether they were sick or well at the end of each trial and checked if they had experienced any disorientation, nausea, or oculomotor symptoms during their debriefing at the end of the experiment. Otherwise the procedure closely followed that of the previous experiment.

#### Participants

Seven female and six male psychology students and staff (mean age = 28 years, *SD* = 6.8 years) from the University of Wollongong participated in this experiment. None of them had participated in Experiment 1. They were all naïve to the experimental hypotheses and reported feeling well at the beginning of the experiment. They had normal or corrected-to-normal vision and no reported existing vestibular or neurological impairments. Ethical approval and participant consent was the same as for Experiment 1.

#### Apparatus

Self-motion simulations were presented on a Razer OSVR Hacker Development Kit HMD. This had a refresh rate of 60 Hz and a resolution of 640 (horizontal) x 800 (vertical) per eye (these stereoscopic stimuli were generated via side-by-side presentation of the left and right eye views on a single 1280 x 800 pixel screen). Self-motion simulations subtended a binocular visual area which was 100 degrees diagonal. During the experimental trials, vection onset latency and vection duration responses were again recorded by pressing a button on the computer’s mouse. The verbal vection strength ratings for each trial were also entered by the experimenter on the computer’s keyboard.

#### Design and procedure

Two independent variables were manipulated in this experiment: (1) Prime Type. Initially these stereoscopic displays simulated that the observer was either stationary (static prime) or oscillating up-and-down as well as from left-to-right (oscillating prime) relative to the 3D cloud of stationary objects. (2) Radial Flow Type. After 10s exposure to the prime, these stereoscopic displays began to simulate self-motion in depth via motion perspective, changing-size and stereoscopic motion cues. This was either smooth forwards self-motion (smooth radial flow) or forwards self-motion combined with simulated horizontal and vertical viewpoint oscillation (oscillating radial flow). There were three blocks of trials—each of which presented all four of the different experimental conditions. The first of these three blocks was treated as practice. On each trial, three different dependent variables were obtained: (1) the vection onset latency (in seconds); (2) the vection strength rating (from 0–10); and (3) whether the participant reported feeling ‘sick’ or ‘well’. As Block did not produce significant vection latency or strength effects in Experiment 1, and we did not obtain FMS ratings in Experiment 2, we did not include Block in the analyses below. During their debriefing at the end of the experiment, we also asked participants: (1) whether they felt sick or well; and (2) whether they were currently experiencing any disorientation, oculomotor or nausea (to confirm that their previous reports of sickness or being well were valid). With the exception of one participant (RM—discussed below), all reported feeling well both throughout and directly after the experiment (no disorientation, nausea or oculomotor symptoms).

### Results

In this HMD-based experiment vection was reported on every trial by all thirteen participants. Of the 156 trials tested, only 7 had vection onsets that occurred during the earlier 10 s priming phase (i.e., less than 5% of trials). As expected the average vection onset latency for all of the conditions tested (*M* = 4.4 s) was shorter than that for Experiment 1 (*M* = 6.2 s). As in the previous experiment, little motion sickness was reported. All 13 participants reported being well throughout the experiment. However, the responses of participant (RM) during debriefing suggested that she was experiencing minor symptoms of disorientation (even though she had consistently reported being “not sick” after each trial). The data for this experiment are also provided as supplementary materials (see S1 Data for Analysis Experiments 1–3).

#### Vection onset latency

**Fig 5:**
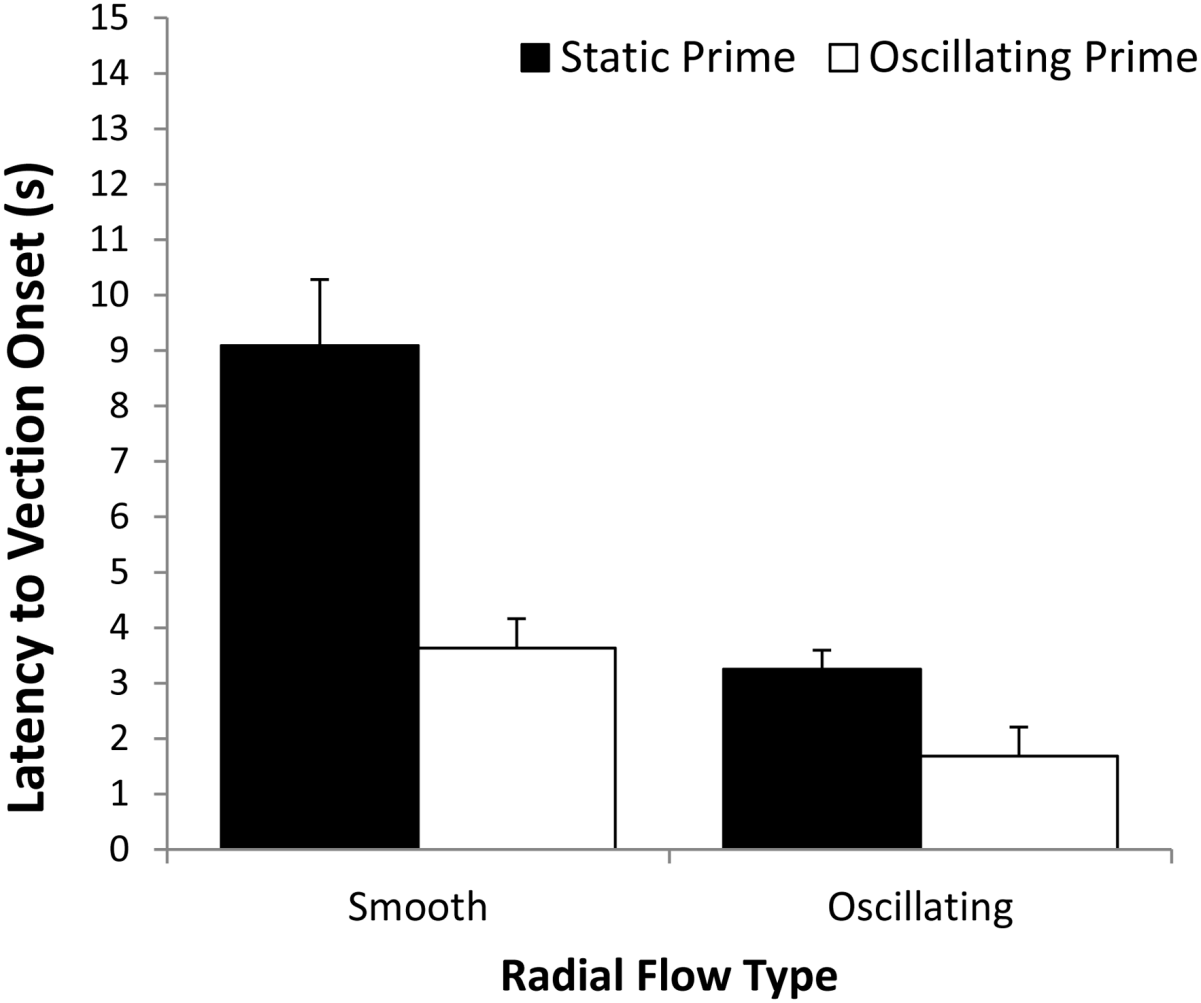
Effects of Prime Type (static or oscillating) on the mean vection onset latencies for smooth and oscillating patterns of radial optic flow. Error bars depict standard errors of the mean (SEMs).

Since it was possible that trials with negative onset latencies represented direct vection induction by prime (rather than a priming effect per se), all negative onset latencies were replaced with zeroes prior to conducting the repeated measures ANOVA. Seven trials (or 4.5% of the total trials) with negative onset latencies were replaced with zeros. Fig 5 shows the adjusted mean vection onset latencies for each of the Prime Type by Radial Flow Type conditions. We found a significant main effect of Prime Type for vection onset latency, *F*(1,12) = 28.246, *p* = 0.0001, partial η^2^ = 0.70 (observed power was 0.998). This indicated that oscillating primes (*M* = 2.7 s) resulted in shorter vection onsets than static primes (*M* = 6.2 s). A significant main effect of Radial Flow Type was also found for vection onset latency, *F*(1,12) = 28.829, *p* = 0.0001, partial η^2^ = 0.71 (observed power was 0.998). This indicated that vection was induced more quickly for oscillating radial flow (*M* = 2.5 s) than for smooth radial flow (*M* = 6.4 s). Unlike Experiment 1, we also found a significant Prime Type by Radial Flow Type interaction, *F*(1,12) = 17.093, *p* = 0.001, partial η^2^ = 0.588 (observed power was 0.966). This indicated that the advantages provided by the oscillating prime were greater for smooth, compared to the oscillating, radial flow. It is likely that this interaction was due to a floor effect for the optimal combined oscillation condition (where the benefits of the oscillating prime were reduced because of the already strong effects of adding oscillation to the large, stereoscopic radial flow display). However, a post-hoc contrast revealed that the oscillating prime followed by oscillating radial flow condition (*M* = 1.7 s, *SD* = 1.9 s) still produced significantly faster vection onsets than of any of the three other conditions tested (*p* < 0.05). A one-way *t*-test on the means of the thirteen participants in this optimal vection condition indicated that their onset latencies were still significantly greater than zero, *t*_12_ = 3.22, *p* = 0.01.

The pattern of results again remained unchanged when these negative onset latencies were removed from the analysis (rather than being replaced by zero as in the above analyses). We calculated the latency data for this more conservative analysis by averaging all of positive latencies across the blocks for each of the condition. There were still significant main effects of Prime Type {*F*(1,12) = 23.92, *p* = 0.0001, partial η^2^ = 0.666} and Radial Flow Type {*F*(1,12) = 28.291, *p* = 0.0001, partial η^2^ = 0.702} for vection onset latency. As in the original analysis above, the Prime Type by Radial Flow Type interaction also remained significant for vection onset latency {*F*(1,12) = 16.409, *p* = 0.002, partial η^2^ = 0.578}.

#### Vection strength ratings

**Fig 6:**
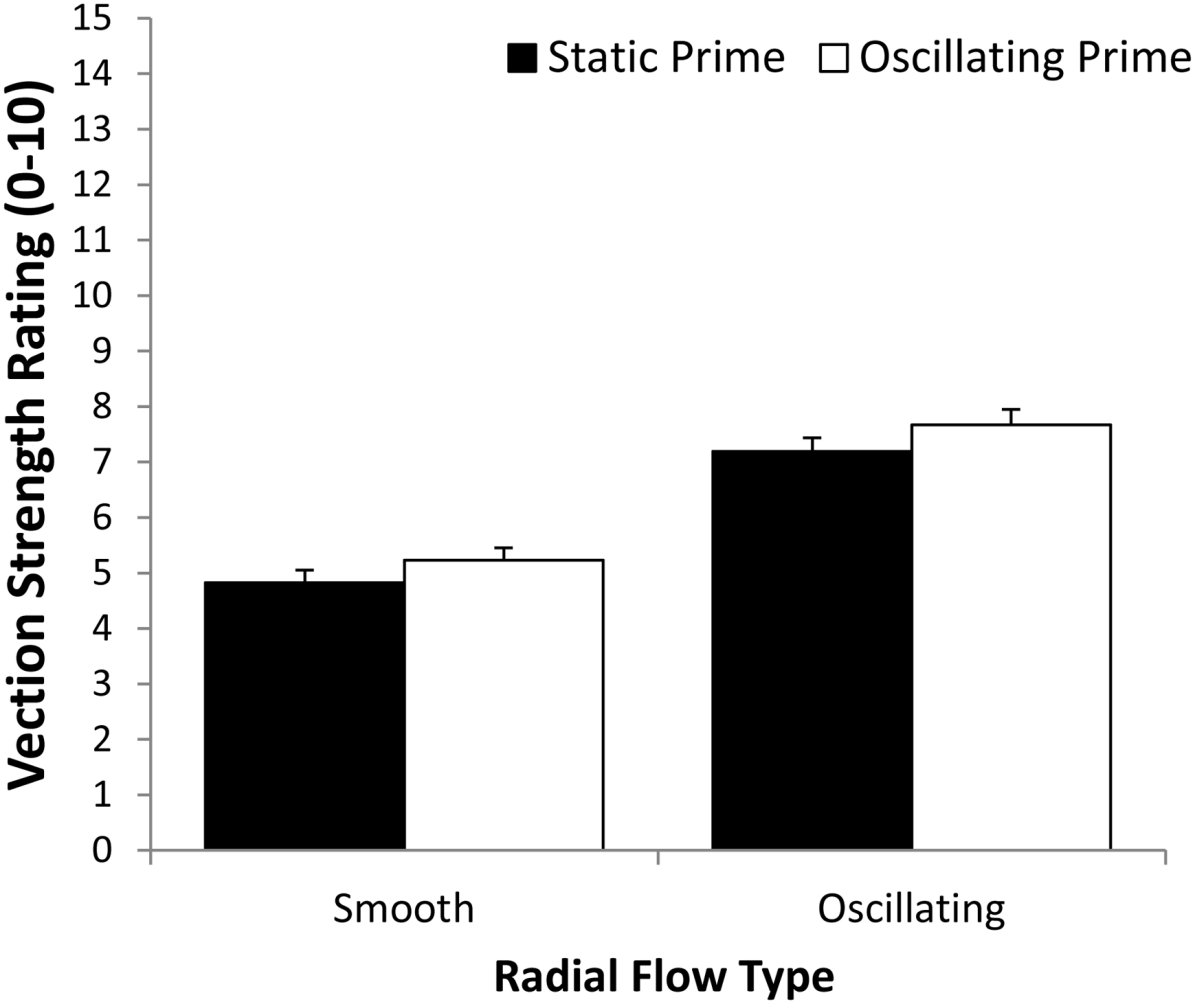
Effects of Prime Type (static or oscillating) on the mean vection strength ratings for smooth and oscillating patterns of radial optic flow. Error bars depict standard errors of the mean (SEMs).

Fig 6 shows the mean vection strength ratings for each of the Prime Type by Radial Flow Type conditions. We found a significant main effect of Prime Type for vection strength ratings, *F*(1,12) = 10.322, *p* = 0.01, partial η^2^ = 0.462. This indicated that oscillating primes (*M* = 6.5) produced stronger vection than static primes (*M* = 6.0). A significant main effect of Radial Flow Type was also found for vection strength ratings, *F*(1,12) = 369.09, *p* = 0.0001, partial η^2^ = 0.969. This indicated that oscillating radial flow (*M* = 7.4) induced stronger vection than smooth radial flow (*M* = 5.0). However, the Prime Type by Radial Flow Type interaction did not reach significance, *F*(1,12) = 0.06, *p* = 0.82, partial η^2^ = 0.005, suggesting that the enhancements to vection strength provided by the two types of oscillation were additive and independent.

## Discussion

The oscillating prime and oscillating radial flow advantages were found to persist for the vection induced in this HMD experiment—even though the field of view was increased and consistent stereoscopic cues were provided for these self-motion simulations. As in Experiment 1, the benefits of oscillation in both trial phases appeared to be additive (although not always independent). Again the optimal condition for vection (in terms of both onset latency and strength ratings) was when the oscillating prime was followed by an oscillating radial flow. The average vection onset latency for this condition was 1.7 s, which was 7.4 s shorter than the average latency for the control condition (i.e., when the static prime was followed by a smooth radial flow).

## Experiment 3: Did the oscillating prime induce vection?

It was possible that 10 s exposures to the oscillating prime were sufficient to induce some horizontal-and-vertical vection on their own (i.e., prior to the 30 s exposures to the smooth and oscillating patterns of radial flow). Consistent with this notion, 4.3% of trials in Experiment 1 and 9% of trials in Experiment 2 which had an oscillating prime were found to produce negative vection onset latencies. This indicates that participants did sometimes report vection when they were exposed to the oscillating prime. Experiment 3 was therefore conducted to further examine whether the oscillating prime was reducing vection onset latencies by priming the observer towards vection or by directly inducing vection. In this control experiment, the horizontal-and-vertical display oscillation (used previously as a vection prime) was presented continuously for 40 s (as opposed to only for 10 s; so as to facilitate vection induction). We compared the vection induced by 40 s of this Oscillating-Only display to that induced by equivalent 40 s presentations of smooth and oscillating patterns of radial flow.

### Methods

The visual motion stimuli and apparatus used here were similar to those in Experiment 2. On each trial, a single optic flow display (either Oscillating-Only, Radial-Only or Oscillating-Radial) was presented continuously for 40 s through the participant’s HMD. The procedure otherwise closely followed that of Experiment 2.

#### Participants

Seven female and five male psychology students and staff (mean age = 32 years, *SD* = 9.3 years) from the University of Wollongong participated in this experiment. Nine of them had previously participated in Experiment 2. The remaining 3 participants had not participated in Experiments 1 and 2. They had also not previously experienced vection in the laboratory. All had normal or corrected-to-normal vision and no reported existing vestibular or neurological impairments. Ethical approval and participant consent were the same as for the previous experiments.

#### Design

One independent variable was manipulated in this experiment: (1) Flow Type. Displays simulated either horizontally-and-vertically oscillating self-motion (Oscillating-Only), smooth forwards self-motion (Radial-Only) or oscillating forwards self-motion (Oscillating-Radial). All three types of display were presented for 40 s (as opposed to either 10 s or 30 s in the previous experiments). There were three blocks—each of which presented all three of the different experimental conditions in a randomized order. As in Experiment 2, the first of these three blocks was treated as practice. Vection onset latencies (in seconds) and vection strength ratings (from 0–10) were obtained for each trial.

### Results

Vection was reported on 98 of the 108 trials tested. Nine of the 10 non-vection trials occurred during Oscillating-Only displays. The only other non-vection trial was found during a Radial-Only display. The 12 participants were each exposed to 3 Oscillating-Only trials. That is, there were 36 Oscillating-Only trials in total. Only 3 (or 8%) of these Oscillating-Only trials were found to have vection onset latencies under 10 s. The data for this experiment are also provided as supplementary materials (see S1 Data for Analysis Experiments 1–3).

#### Vection onset latency

**Fig 7:**
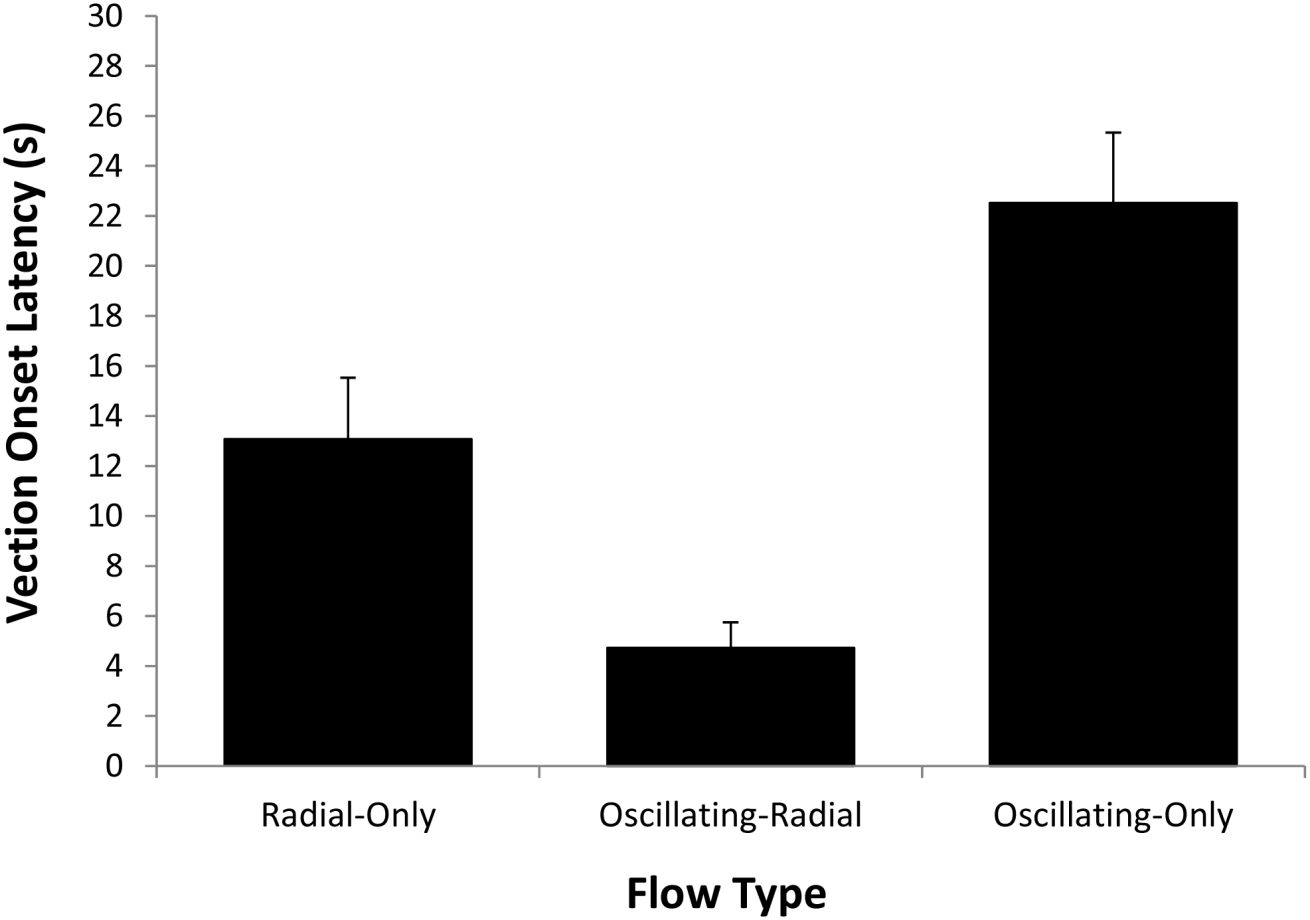
Effects of Flow Type (Radial-Only, Oscillating-Radial, Oscillating-Only) on mean vection onset latencies. Error bars depict standard errors of the mean (SEMs).

Trials where vection was not induced were assigned a vection onset latency of 41 s (i.e., the 40 s duration of the trial plus an additional 1 s). Fig 7 shows the mean vection onset latencies for each of the three different Flow Type conditions. We found a significant main effect of Flow Type for vection onset latency, *F*(2,22) = 31.02, *p* = 0.0001, partial η^2^ = 0.74. Pairwise comparisons revealed that: (1) Oscillating-Only flow (*M* = 22.5 s; *SD* = 9.8 s) produced significantly longer vection onsets than either the Radial-Only (*M* = 13.07 s; *SD* = 8.5 s) or the Oscillating-Radial (*M* = 4.72 s; *SD* = 3.5 s) flow; and (2) vection onset latencies were significantly shorter for the Oscillating-Radial flow than for the Radial-Only flow (all *p*’s < 0.05 and they remained so after Bonferroni correction).

#### Vection strength ratings

**Fig 8:**
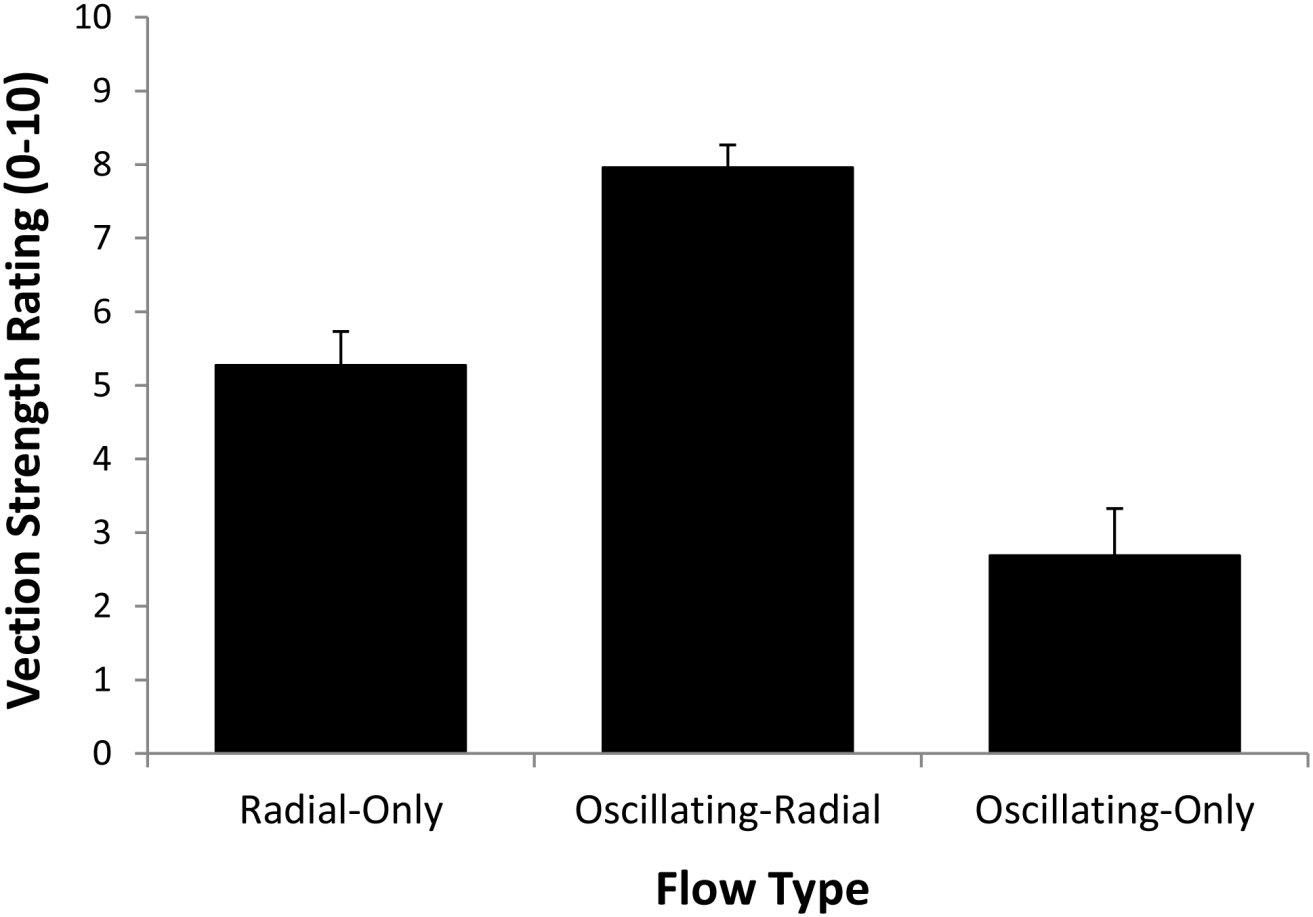
Effects of Flow Type (Radial-Only, Oscillating-Radial, Oscillating-Only) on mean vection strength ratings. Error bars depict standard errors of the mean (SEMs).

Fig 8 shows the mean vection strength ratings for each of the three different Flow Type conditions. We found a significant main effect of Flow Type for vection strength ratings, *F*(2,22) = 78.05, *p* = 0.0001, partial η^2^ = 0.87. Pairwise comparisons revealed that: (1) Oscillating-Only flow (*M* = 2.69) produced significantly weaker vection ratings than either the Radial-Only (*M* = 5.27) or the Oscillating-Radial (*M* = 7.96 s) flow; and (2) vection ratings were significantly stronger for the Oscillating-Radial flow than for the Radial-Only flow (all *p*’s < 0.05 and they remained so after Bonferroni correction).

## Discussion

While most Oscillating-Only trials were found to induce vection when display durations were increased to 40 s, it was weaker than the vection induced by Radial-Only flow, which in turn was weaker than the vection induced by Oscillating-Radial flow. It also took considerably longer to induce vection with this Oscillating-Only flow. The mean vection onset latency for these Oscillating-Only trials was 22.5 s (with a standard deviation of 9.8 s). This average onset latency was more than twice the presentation duration of the priming stimuli in Experiments 1 and 2. These findings suggest that while 10 s presentations of Oscillating-Only displays might have occasionally induced weak vection in Experiments 1 and 2, this vection was not induced during the priming phase of most trials. Instead, the evidence suggests that the Oscillating-Only display served mainly as a vection prime (as opposed to a vection inducer) in most of the trials in these experiments.

## General discussion

This study found that vection onset latencies could be substantially reduced by presenting participants with an oscillating (as opposed to a static) visual priming stimulus for just 10 seconds. However, these vection onset latencies could be still further reduced by also adding the same visual oscillation to the subsequently presented self-motion simulation as well. Similar findings were observed in both the large screen projection and HMD experiments, suggesting that these benefits might generalise to other methods and types of self-motion simulation (however this must of course still be confirmed by further research). In Experiment 1, non-stereoscopic self-motion simulations were presented on a large external display. Under these conditions, combining both types of oscillation was found to reduce vection onset latencies by 45% on average (down to 4.6 s from a mean latency of 8.3 s in the no oscillation control condition). In Experiment 2, participants viewed stereoscopic versions of these self-motion simulations through a wide field of view HMD. Under these conditions, combining the two types of oscillation was found to reduce vection onset latencies by an impressive 81% (down to 1.7 s from a mean latency of 9.1 s in the no oscillation control condition). This latter finding suggests that instantaneous vection may indeed be possible. In the past, there have been only anecdotal reports that it can occur in highly compelling and immersive environments, such as Ian Howard’s tumbling room [74].

In the introduction we outlined several perceptual reasons why an oscillating visual prime might be expected to reduce vection onset latency. However, in any vection experiment there is always the possibility that cognitions, experimental demands, or other extraneous factors might influence vection reporting behaviour as well [7,9]. So before discussing the current findings in the light of these proposed perceptual mechanisms, one must first rule out non-perceptual causes of these vection effects. It has been previously established that the vection advantage for oscillating radial flow (replicated here) is independent of both experimenter demands and participant cognitions [4,75]. In fact, the default expectations of naïve participants are that these visually simulated accelerations should impair (not enhance) the induction of illusory self-motion. Thus we will restrict our discussion here to non-perceptual explanations for the effects of the oscillating prime. In this study, different groups of naïve participants were tested in Experiments 1 and 2, which showed very similar effects of viewpoint oscillation on vection onset latency. In order to minimise experimental demands, participants were simply told that they should press a button whenever they experienced self-motion and hold it down as long as that experience continued. We did not provide specific instructions that they should press the button during a particular phase of the trial or when they experienced self-motion in a particular direction. Even so, 96–98% of vection onset latency responses occurred in the second (radial flow) phase of these trials (across all conditions tested). While the oscillations presented in both trial phases were potentially consistent with self-motion, participants rarely pressed the button when viewing an oscillating prime. Based on their feedback given during debriefing, a few participants did press the button when they experienced a weak perception of horizontal-and-vertical self-oscillation during these priming displays (discussed further later on). Thus the available evidence suggests that the vection onset responding of our participants was genuine. Another possibility was that accidental button pressing might have artificially reduced our participants’ vection onset latencies. However, this possible artefact was removed by classifying vection onset latency (in all experiments) as the time of the first button press for each trial which lasted at least 1 s.

Based on the reasons outlined above, we therefore conclude that the effects of visual oscillation on vection were perceptual in origin in the current study. Earlier, in the introduction, we provided three possible perceptual reasons why prior exposure to an oscillating visual prime might reduce vection onset latencies. While Experiments 1 and 2 were not designed to specifically test these different hypotheses, we will examine how well each of these hypotheses fits their data and also the data obtained in control Experiment 3.

The first possibility was that the oscillating prime might have induced some horizontal and vertical vection by itself (prior to the participant’s exposure to either the smooth or oscillating forwards self-motion display). Previously it has been shown that simulated horizontal and vertical viewpoint jitter can induce weak vection when presented on its own [69]. In a similar fashion, it appears that the oscillating primes used in the current study sometimes also induced weak vection (based on our participants’ button pressing responses, their comments during debriefing and the findings of control Experiment 3). It is possible that this weak preliminary vection contributed to the current oscillation effects found for vection onset latency and vection strength. However, since only 4.3–9% of oscillating prime trials induced weak vection during the initial 10 s, this does not appear to have been the main cause of the latency reduction. Experiment 3 showed that these Oscillating-Only stimuli required on average 22.5 s to reliably induce vection (more than twice the 10 s duration of the prime presentation). The occurrence of weak preliminary vection also cannot explain why vection onset latency was further reduced by providing oscillation in both priming and radial flow trial phases (compared to when oscillation was only present during priming phase). Moreover, Ito [71] has shown that a change in the simulated self-motion direction consistently produces vection drop-outs. Thus, even when vection was occasionally induced by the oscillating prime, it was unlikely that this weak (left-right and up-down) oscillatory vection would have transferred directly to the subsequently simulated forwards self-motion. Instead this vection should have dropped out as the trial transitioned from the initial oscillatory priming phase to the radial flow phase. This further supports our proposal that the Oscillating-Only displays served mainly as vection primers (rather than vection inducers) in Experiments 1 and 2.

The second possibility was that prior exposure to the simulated viewpoint oscillation might have triggered a sensory readjustment in our stationary observers that favoured vection induction. While the vestibular information was consistent with either being stationary or moving at a constant velocity, the visual oscillation used in this experiment always simulated self-acceleration (not just self-motion). It was proposed that prolonged exposure to an oscillating prime stimulus would cause the conflicting vestibular inputs to be down-weighted (in favour of the visual information indicating self-motion). Reducing the contribution of this vestibular information to perceptions of self-motion should therefore increase the vection inducing potential of any subsequently presented optic flow (irrespective of whether it was smooth or oscillating). However, vestibular down-weighting might also be able to explain why the oscillating prime produced greater benefits for oscillating (compared to smooth) patterns of radial flow. Providing oscillation during the radial flow trial phase might have prolonged this vestibular down-weighting and even intensified its vection enhancing effects (as indicated by faster vection onsets and stronger vection ratings). Thus, the current findings of Experiments 1 and 2 both seem to be generally consistent with this sensory readjustment proposal.

The third possibility was that prior exposure to a globally oscillating visual prime might alter the observer’s subsequent sensitivity to self-motion consistent radial flow. According to the proposal, this increased sensitivity to global patterns of optical flow might have also been preserved/strengthened by continuing to provide visual oscillation during the radial flow phase of the experiment as well. While the oscillating primes used in the current study were always globally coherent oscillations (i.e., simulated viewpoint oscillation), it might be possible to produce similar priming benefits for vection with different types of display motion. Recently a poster by Ni et al. [76] reported that presenting global patterns of random motion before a self-motion display also reduced vection onset latency. Their preliminary report at a recent conference appears to corroborate our findings—even though they used globally random, as opposed to globally coherent, motions as their vection priming stimuli. It is however also possible that the mechanism underlying Ni et al.’s [76] intriguing priming effect might be somewhat different to the effects of our own global motion prime. Instead of their prime directly sensitizing the observer to self-motion consistent patterns of optic flow, prolonged exposure to its random motions might have adapted the observer’s local motion processing. This could have indirectly favoured the perception of global motion from optic flow and therefore led to quicker vection induction.

As noted in the introduction, this study itself was not designed to discriminate between these three possible perceptual explanations of visual priming effects on vection. As this type of visual priming has not been examined before, our main purpose was to show that it can improve vection and that these priming benefits do generalise to different display systems. While the available evidence suggests that direct vection induction by the prime was unlikely to account for the current priming findings, they do not clearly favour one of the two remaining hypotheses over the other (i.e., sensory readjustment and increased sensitization to global optical flow). Future studies will be required to more systematically test the predictions of these different accounts and determine the underlying mechanisms of these oscillation-based advantages.

However, even without knowing mechanisms responsible, there should still be a number of practical applications of these findings. For example, a videogame developer might want gamers to feel they are moving forward as quickly as possible in their game to provide a more compelling and immersive user experience. Similarly, designers of an immersive architecture walkthrough or telepresence/telerobotics application might want their users to experience a natural and compelling sensation of being in and moving through the virtual or remote space to provide a close-to-real user experience, and facilitate spatial orientation [7,25]. In such situations it would not matter to the developer if the briefly presented oscillatory prime (which mimics minimal head-bobbing) induced weak illusory self-motion in users (as their perceived left-right and up-down self-motions would be oscillatory in nature, and therefore, on average their perceived position in 3-D space would not change until the simulated forward self-motion began). As noted above, we believe that this is the least likely of the three proposed hypotheses to explain the effects of the oscillating prime. Similarly, the developer would also not need to know whether sensory readjustment or increased sensitization to global flow was responsible for the benefits of the oscillating prime in order to take advantage of them.

## Conclusions

The current study showed that it was possible to significantly reduce vection onset latencies and enhance vection strength with purely visual display manipulations (i.e., adding simulated viewpoint oscillation before and during the visually simulated self-motion). The simulated viewpoint oscillation used in these experiments was not found to significantly increase either the likelihood or the severity of motion sickness, and participants did not report any other adverse side-effects related to the oscillating prime. However, it should be noted that our exposures to display motion were quite brief and that sickness severity ratings did increase with repeated exposures, as is common for vection and VR studies. Past studies have also shown that higher frequencies of simulated viewpoint oscillation can led to increased motion sickness (e.g., [77]). Thus, while the current findings appear promising for the future use this type of display manipulation in VR and simulation based applications, some caution in their implementation is still warranted. As the likelihood of sickness increases with exposure time to display motion, one possible solution might be to only provide these (potentially more provocative) visual oscillations during the short initial priming phase (not during the subsequent and typically much longer self-motion simulation phase).

In our study providing an oscillating prime that lasted only 10s was enough to reduce vection onset latencies by up to 71% on average (or 81% when the same visual oscillation was also added to the self-motion simulation as well). These reductions in vection onset latency are much larger than have been previously observed when visual self-motion simulations have been complemented by adding accurate or metaphorical auditory self-motion cues [78–83], observer vibrations [26,44,84], air flow to the observer’s face [85], consistent biomechanical walking cues (via a circular treadmill) [86] or physical motion cueing (via a wheelchair interface or a gaming chair) [23,87]. The reduction in vection onset latency produced by our oscillating prime appeared to be comparable to the effects of providing small physical motion cues to observers with a motion platform [31,42,44], but they were achieved with considerably less expense and without any need for additional equipment. We are currently investigating how to further improve the effectiveness of this oscillating prime, which might ultimately allow us to cost-effectively provide compelling and (almost) instantaneous self-motion perception in a wide variety of application areas (including tele-presence, tele-robotics, virtual reality, vehicle simulation, architecture walk-throughs and entertainment) [14,26].

## Supporting information

S1 Demo Movie A video of the static prime followed by smooth radial flow (MP4). This screen capture recording was created using the Game DVR app in windows 10. Please note that speed will be approximate due to differences in frame rate and screen size.
(MP4)

S2 Demo Movie A video of the static prime stimulus followed by oscillating radial flow (MP4). This screen capture recording was created using the Game DVR app in windows 10. Please note that speed will be approximate due to differences in frame rate and screen size.
(MP4)

S3 Demo Movie A video of the oscillating prime stimulus followed by smooth radial flow (MP4). This screen capture recording was created using the Game DVR app in windows 10. Please note that speed will be approximate due to differences in frame rate and screen size.
(MP4)

S4 Demo Movie A video of the oscillating prime stimulus followed by oscillating radial flow (MP4). This screen capture recording was created using the Game DVR app in windows 10. Please note that speed will be approximate due to differences in frame rate and screen size.
(MP4)

S1 Data for Analysis Experiments 1–3 This excel spreadsheet (.xlxs) provides the vection latency, strength and sickness data for Experiment 1 (vection with large external display), Experiment 2 (vection with HMD), and control Experiment 3 (vection with oscillating prime). The data for these experiments are each presented on separate Excel worksheets.
(XLSX)
